# *QuickStats*: Injury Deaths* as a Percentage of Total Deaths, by Age Group — National Vital Statistics System, United States, 2019

**DOI:** 10.15585/mmwr.mm7031a3

**Published:** 2021-08-06

**Figure:**
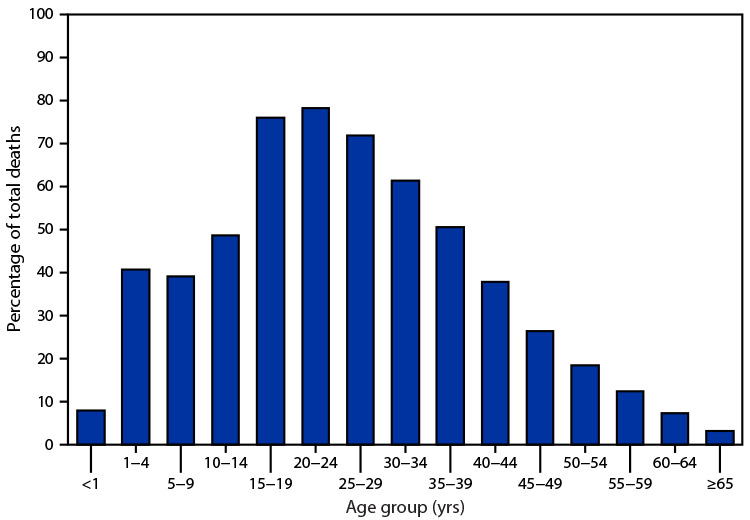
Injuries accounted for the majority of deaths among persons aged 15–39 years, with the highest percentages among those aged 15–19 (76.0%) and 20–24 years (78.2%). The percentage of injury deaths was lowest among those aged <1 year (7.9%), 60–64 years (7.5%), and ≥65 years (3.4%).

**Source:** National Center for Health Statistics, National Vital Statistics System, Mortality Data, 2019. https://www.cdc.gov/nchs/nvss/deaths.htm

For more information on this topic, CDC recommends the following link: https://www.cdc.gov/injury

